# Use of Ice in Exhausting Diseases

**Published:** 1848-06

**Authors:** 


*Use of Ice in Exhausting Diseases.*—Some interesting cases are quoted in a recent number of the *Revue Medico-Chirurgicale*, from a French journal, in which ice taken internally seemed to be of great service in reviving powers fast sinking. The writer employs it in very various diseased conditions, providing these manifest the signs of intense debility. The reaction it induces may prove curative in some cases, while in others, in which this is impossible, a marked temporary amelioration of the patient's state occurs. In the cases in question there is great atony and extenuation, and an extreme aversion to any food whatever, with or without a development of heat. A number of morbid states and organic lesions, having no other points in common, may induce this condition. Iced water does not succeed anything like so well as the administration of the ice in little lumps, which by requiring time for their solution ensure its gradual introduction. These impart great tone to the system, and revive the inclination for food in a remarkable manner.—*Rev. Med. Chir.*—*Ib.*

